# SCRAMBLE’N’GAMBLE: a tool for fast and facile generation of random data for statistical evaluation of QSAR models

**DOI:** 10.1007/s11696-017-0215-7

**Published:** 2017-06-05

**Authors:** Piotr F. J. Lipiński, Przemysław Szurmak

**Affiliations:** 1 Department of Neuropeptides, Mossakowski Medical Research Centre Polish Academy of Sciences, 02-106 Warsaw, Poland; 2 ChemPharmSoft, 01-926 Warsaw, Poland

**Keywords:** *y*-Randomization, Chance correlations, QSAR, QSAR validation

## Abstract

A common practice in modern QSAR modelling is to derive models by variable selection methods working on large descriptor pools. As pointed out previously, this is intrinsically burdened with the risk of finding random correlations. Therefore it is desirable to perform tests showing the performance of models built on random data. In this contribution, we introduce a simple and freely available software tool SCRAMBLE’N’GAMBLE that is aimed at facilitating data preparation for *y*-randomization and pseudo-descriptors tests. Then, four close-to-real-world modelling situations are analysed. The tests indicate what the quality of obtained QSAR models is like in comparison to chance models derived from random data. The non-randomness is not the only requirement for a good QSAR model, however, it is a good practice to consider it together with internal statistical parameters and possible physical interpretations of a model.

**Electronic supplementary material:**

The online version of this article (doi:10.1007/s11696-017-0215-7) contains supplementary material, which is available to authorized users.

## Introduction

Quantitative Structure-Activity Relationship (QSAR) modelling is an important field of research in current medicinal chemistry. QSAR models relate the structure of chemical compounds to their biological activities:

$${\text{activity = }}f ( {\text{structure)}} .$$

The aim of building such models is to explain and/or to predict the activity of a group of compounds and thus to facilitate and direct search for new active substances.

In QSAR, the structure of a chemical compound is represented mathematically by molecular descriptors. These can be based on physicochemical properties measured experimentally (e.g. partition coefficient LogP), quantities calculated by quantum chemistry methods (e.g. HOMO/LUMO energies) (Karelson et al. 1996) or be derived from other theoretical bases (e.g. chemical graph theory, (Balaban 1985; Helguera et al. 2008) theory of quantitative chirality (Ostrowski et al. 2012; Jamróz et al. 2012 etc.). The number of currently available descriptors is enormous (Dearden 2016). There are several applications designed specifically at their calculation [for example DRAGON by Talete Srl that computes ca. 5000 descriptors (Talete Srl 2010)] and such a functionality is present in probably all drug design and discovery suites like Accelrys Discovery Studio (Accelrys Software Inc. 2009), Schrödinger Suite (2017), molecular operating environment (Chemical Computing Group ULC 2017) to mention only a few.

In a typical situation a researcher has at his or her disposal a scarce number of compounds with determined activity (like 20 to several dozen) and an alluring plenitude of molecular descriptors (hundreds or thousands) to be used for constructing QSAR equations. This makes the danger of overfitting data a very likely one.

The common statistical parameters, like coefficient of determination, standard deviation, significance etc. are not able to discern ‘good’ models from overfitted ones (Rücker et al. 2007). This cannot be also done by any kind of internal validation procedures, like leave-one-out, leave-many-out etc. An ultimate test of validity and utility of a given QSAR model is always the external validation on an independent, large enough, properly designed set of new derivatives (Gramatica 2007). This is, however, rarely possible due to the lack of resources and/or time. In such circumstances, perhaps the only affordable way to see if studied QSAR models work better than the pure chance is to simulate the ‘predictive power’ of the pure chance. Two tests could be of help here: *y*-scrambling and pseudo-descriptors test (Clark et al. 2001; Rücker et al. 2007).

The *y*-scrambling (*y*-randomization, response randomization) is a form of a permutation test, where the values of the response variable (*y*) are randomly ascribed (scrambled) to different compounds, while the descriptors values (*x’s*) are left intact. Scrambled data are then used for training QSAR models. In the pseudo-descriptors test, the descriptors (*x’s*) are replaced by random numbers (pseudo-descriptors) that are also subsequently used to train QSAR equations.

Both tests are run over several to several dozen times, and from each run best coefficient of determination *r* ^2^, leave-one-out cross-validation correlation coefficient *q* ^2^ and perhaps other adequate statistical parameters are collected. The mean highest *r* ^2^ (mhr^2^) and *q* ^2^ (mhq^2^) along with their standard deviations (SD) are calculated. This allows to assess the ‘predictive power’ of the pure chance, and the truly good models should have their *r* ^2^ and *q* ^2^ significantly better than this.

Unfortunately, these simple tests are very often not included into QSAR studies. One of the reasons, apart from their time-consuming character, might be in a difficulty in obtaining random data for simulations. Not every researcher is enough computer proficient to generate them on his own, and not everyone has access to good statistical software that could accomplish this without much trouble. The software, in majority, if not in all cases, is also not suited to working with common formats of chemical table files like SDF (Dalby et al. 1992) that are usually accepted by QSAR modelling software. The need for manual operations on numerous, large spreadsheets of numbers and chemical files can be an actual obstacle, and discouraged researchers omit these insightful tests.

In order to facilitate data preparation for the tests, a simple and free software tool SCRAMBLE’N’GAMBLE is proposed. It is a stand-alone Java application with both graphic user interface as well as a command-line manageability. SCRAMBLE’N’GAMBLE reads in comma-separated files (csv) and chemical table files by MDL (sdf) containing descriptors and activity data. It can perform y-scrambling as well as generate pseudo-descriptors given number of times and output the results into a csv file, but also directly into a sdf file immediately usable in most QSAR programs. SCRAMBLE’N’GAMBLE is available free of charge at: http://www.drugdesign.pl/scramble-n-gamble/.

In order to demonstrate the importance of simulating random chance performance along with building QSAR models, let us expose the following Cases: I. classical QSAR modelling (descriptors based on 2D structures) of steroids’ affinity for the sex-hormone-binding globulin, II. classical QSAR modelling (descriptors based on 2D and 3D structures) of steroids’ affinity for the corticosteroid-binding globulin, III. Fujita-Ban QSAR modelling of the effective dose of some fentanyls in the mouse hot plate test and IV. a classification model for discerning glucocorticoid receptor binders and non-binders.

## Experimental

### Molecules, activity data, descriptor calculation and modelling procedure

In all Cases, a general workflow was as follows. First, molecules with activity data for a given molecular target were collected and divided into a training set and a test set. Second, molecular descriptors were calculated. Constant and near-constant descriptors were deleted from the pool, and further reduction was done by checking intercorrelations between descriptors. In pairs where the coefficient of correlation was larger than 0.90, one of the descriptors was randomly excluded. Third, QSAR models were trained. Fourth, random data for *y*-scrambling and pseudo-descriptors test were generated using SCRAMBLE’N’GAMBLE and the tests were performed by training QSAR models in the same way as the ones based on true data were trained. Fifth, the performance of the latter was checked on test sets.

Details of the workflow for singular Cases are given in Table 1.

**Table 1:** Details of the workflow for singular cases

Case	I	II	III	IV
QSAR task	Linear equation of up to 3 variables, descriptors based on 2D structure	Linear equation of up to 3 variables, descriptors based on 3D structure	Linear equation, Fujita-Ban model (Fujita and Ban 1971)	Classification model
Molecules	Various steroids (Fig. 2)	Various steroids (Fig. 2)	Fentanyl derivatives (3-methyl-1,4-disubstituted piperidines) (Table SI-1 in Electronic supporting material)	Various compounds (Tables SI-5 to SI-8)
Dependent variable	Logarithm of binding affinity to sex-hormone-binding globulin (SHBG) (Fig. 2)	Logarithm of binding affinity to corticosteroid-binding globulin (CBG) (Fig. 2)	Effective dose ED_50_ in mouse hot plate test (analgesic activity test) (Table SI-1)	Whether a molecule binds or does not bind the glucocorticoid receptor (Tables SI-5 to SI-8)
Training set	21 steroids of the benchmark Cramer data set (Cramer et al. 1988; Coats 1998) (S1-S21 in Fig. 2)	21 steroids of the benchmark Cramer data set (Cramer et al. 1988; Coats 1998), chosen by clustering from 31 molecules: S2, S3, S4, S5, S7, S8, S9, S10, S11, S14, S15, S18, S19, S20, S22, S24, S25, S26, S28, S29, S31 in Fig. 2	36 active derivatives (Lalinde et al. 1990) (Table SI-1)	100 active molecules and 3600 decoys randomly chosen from Directory of useful decoys, enhanced (DUD-E). (Mysinger et al. 2012) (Tables SI-5 and Table SI-7)
Test set	Up to 12 molecules (within the applicability domains of the models found) taken from the extended benchmark steroid data set (Cherkasov et al. 2008) (S32-S44 in Fig. 2)	10 steroids of the benchmark Cramer data set (Cramer et al. 1988; Coats 1998), chosen by clustering from 31 molecules: S1, S6, S12, S13, S16, S17, S21, S23, S27, S30 in Fig. 2	10 inactive derivatives (Lalinde et al. 1990) (Table SI-1)	Other 50 active molecules and 1800 decoys randomly chosen from Directory of Useful Decoys, Enhanced (DUD-E). (Mysinger et al. 2012) (Tables SI-6 and Table SI-8)
Calculation of molecular descriptors	2D descriptors in DRAGON 6 (Talete Srl 2010)	Structure optimization (B3LYP/6-31G*) in Gaussian 09 (Frisch et al. 2009) (full citation in Electronic supporting material); vibrational frequencies to check imaginary frequencies; atomic charges (q-descriptors) calculated using the CHELPG algorithm (Breneman and Wiberg 1990); 2D and 3D molecular descriptors in Accelrys Discovery Studio (Accelrys Software Inc. 2009); Sinister-Rectus Chirality Measures (^SR^CMs) (Ostrowski et al. 2012; Jamróz et al. 2012; Ostrowski et al. 2013) and Continuous Chirality Measures (CCMs) (Zabrodsky and Avnir 1995), ^SR^CMs calculated using the CHIMEA software (Jamróz 2010) available at http://www.smmg.pl, while CCMs using a web page from Hebrew University of Jerusalem (Zayit et al. 2011)	Indicator variables	2D descriptors in DRAGON 6 (Talete Srl 2010)
Descriptors calculated	3764	127	9	3764
Descriptors included in training the models	89	49	9	1090
Training QSAR model	Genetic function approximation (GFA) algorithm (Rogers and Hopfinger 1994) in Discovery Studio, GFA settings were as default	Genetic function approximation (GFA) algorithm (Rogers and Hopfinger 1994) in Discovery Studio, GFA settings were as default	Linear regression routine in Microsoft Excel 2016	In-house script based on a Python scikit-learn library for machine learning. (http://scikit-learn.org/); the depth of the tree was set to be maximally 3
Number of random data sets generated	300 (*y*-scrambling, pseudo-descriptors with original distributions, pseudo-descriptors with uniform distributions)	300 (*y*-scrambling, pseudo-descriptors with original distributions, pseudo-descriptors with uniform distributions)	300 (*y*-scrambling, pseudo-descriptors with original distributions, pseudo-descriptors with uniform distributions)	25 (pseudo-descriptors with original distributions, pseudo-descriptors with uniform distributions)

### Evaluation of regression models

For regression models in Cases I and II, standard statistical metrics were applied. These are:

*r* ^2^ coefficient of determination in the training set,

*q* ^2^ cross-validated coefficient of determination in the training set (internal validation, leave-one-out procedure)

*R* ^2^ coefficient of determination in the test set (external validation).

The *r* ^2^ and *q* ^2^ values were compared to mean highest *r* ^2^ (mhr^2^) and *q* ^2^ (mhq^2^) from y-scrambling and pseudo-descriptors tests in order to check whether the models perform better than chance models.

Additionally, ${}^{c}R_{p}^{2}$ and $r_{{m \left( {\text{test}} \right)}}^{2}$ parameters were applied calculated as proposed by the Roy group (Pratim Roy et al. 2009; Mitra et al. 2010). Both these metrics should be greater than 0.5 for an acceptable model. The fulfilment of this criterion with regard to $r_{{m \left( {\text{test}} \right)}}^{2}$ parameter ensures that a model predicts the exact values of the response data. High ${}^{c}R_{p}^{2}$ values allow to consider a model to be robust and not just the outcome of a chance correlation.

For the models in Case II, additional parameters were checked. First, the internal cross-validation was performed also in leave-three-out procedure, giving $q_{(L3O)}^{2}$—a cross-validated coefficient of determination in the training set (leave-three-out). Furthermore, another type of randomization experiment was performed (Wold et al. 1998). Here *y*-scrambled data (25 runs) were used to refit the Case II models. The obtained *r* ^2^ and *q* ^2^ values were then plotted against the correlation coefficients of original *y* and permuted *y* data. The resulting intercepts ($R_{\text{int}}^{2}$ and $Q_{\text{int}}^{2}$) are expected to be below 0.4 and 0.05 respectively for valid models.

### Evaluation of decision trees

For all decision trees (Case IV) the number of True Positives (TP), True Negatives (TN), False Positives (FP) and False Negatives (FN) was collected. The following metrics were used for assessment of the decision trees: accuracy (ACC), precision (PREC), sensitivity (SENS), specificity (SPEC), fall-out (FALL) and F1-score (F1). They are given by the expressions:

1 $${\text{ACC = }}\frac{\text{TP + TN}}{\text{TP + TN + FP + FN}}$$

2 $${\text{PREC = }}\frac{\text{TP}}{\text{TP + FP}}$$

3 $${\text{SENS = }}\frac{\text{TP}}{\text{TP + FN}}$$

4 $${\text{SPEC = }}\frac{\text{TN}}{\text{TN + FP}}$$

5 $${\rm{FALL = 1}} - {\rm{SPEC}}$$

6 $${\text{F1 = }}\frac{{ 2 {\text{TP}}}}{{ 2 {\text{TP + FP + FN}}}}{ = 2} \times \frac{{{\text{PREC}} \times {\text{SENS}}}}{\text{PREC + SENS}}.$$

## Results and discussion

### Software description

**Fig. 1:**
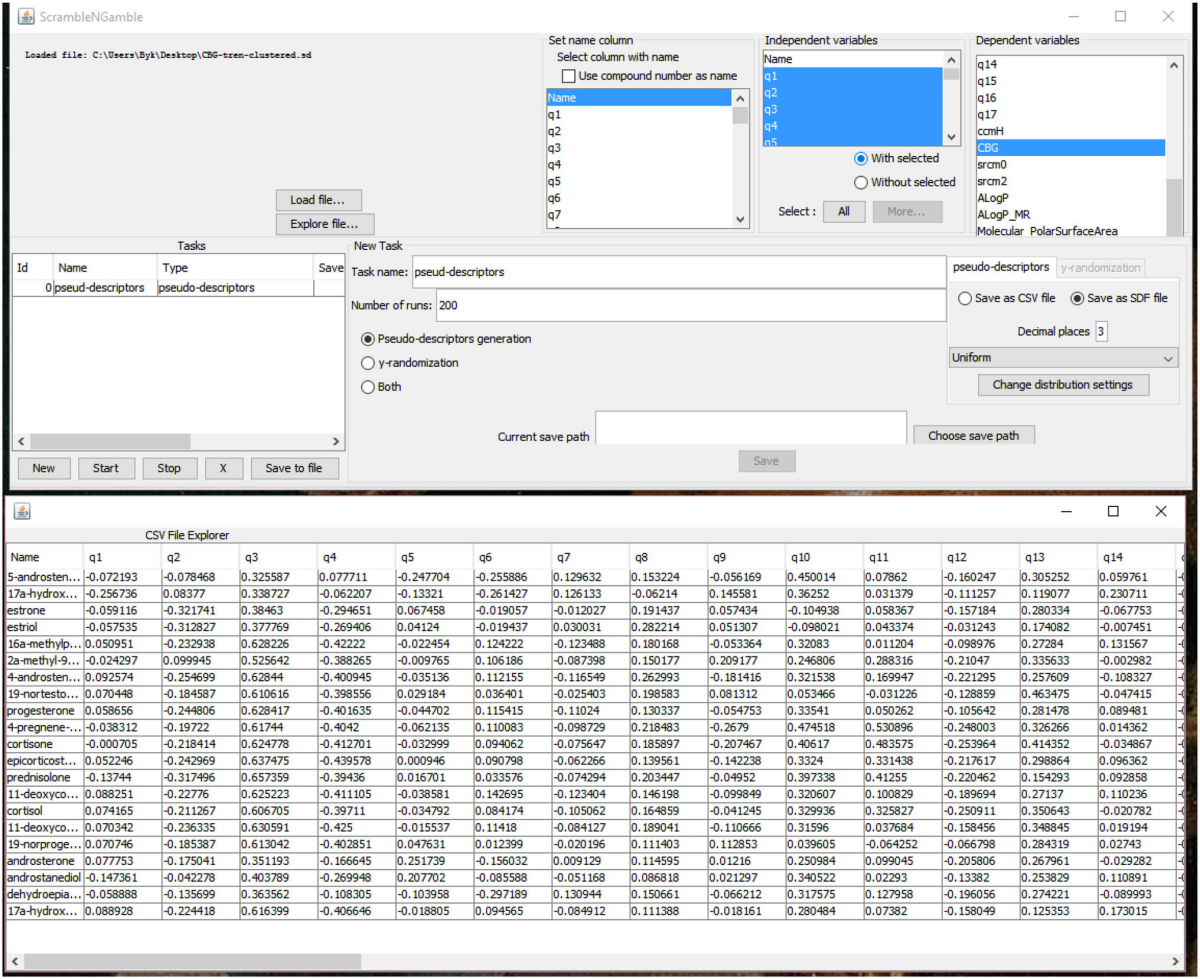
General view of SCRAMBLE’N’GAMBLE interface

SCRAMBLE’N’GAMBLE is a fast and user-friendly software for generation of random data for the purposes of QSAR model validation. The program can read and output both comma-separated files (csv) as well as chemical table files by MDL (sdf) containing molecular descriptors and activity data. Upon selecting which fields should be scrambled or replaced with random data (pseudo-descriptors), the user is able to obtain a required number of randomized data sets in csv or sdf files. The latter are most often accepted by QSAR modelling software. SCRAMBLE’N’GAMBLE may be run in a graphic user interface mode (Fig. 1), but it is also manageable in the command-line mode.

The generation of random (or to be said more precisely: pseudo-random) numbers is achieved using Mersenne Twister 19937 generator (Matsumoto and Nishimura 1998) implemented in UncommonMaths Java library (Dyer 2006). The generator has been shown to generate high quality random numbers and pass many statistical tests for randomness. It is possible to select a distribution from which random numbers will be generated: uniform, normal, binomial, Poisson or exponential. The user may also want to keep original distributions of variables, and in such case the program will perform *x*-scrambling. SCRAMBLE’N’GAMBLE is available free of charge at: http://www.drugdesign.pl/scramble-n-gamble/.

The examples and importance of performing random data tests in QSAR validation are provided by considering four close-to-real-world modelling situations.

### Case I

Sex-hormone-binding globulin (SHBG) is a transport glycoprotein produced in all vertebrates except for birds. SHBG binds preferentially sex hormones (androgens and oestrogens) in the bloodstream and in this way it has impact on the concentration of their free, supposedly biologically active, fractions. Its role in various endocrine disorders is well described (Anderson 1974; Cunningham et al. 1983; Key et al. 2002; Hammond 2011; Caldwell and Jirikowski 2014). Environmental toxicology points also to the importance of SHBG in the endocrine disruption in men and animals caused by exogenous substances (Wilson et al. 2007; Saxena et al. 2014; Hong et al. 2015).

**Fig. 2:**
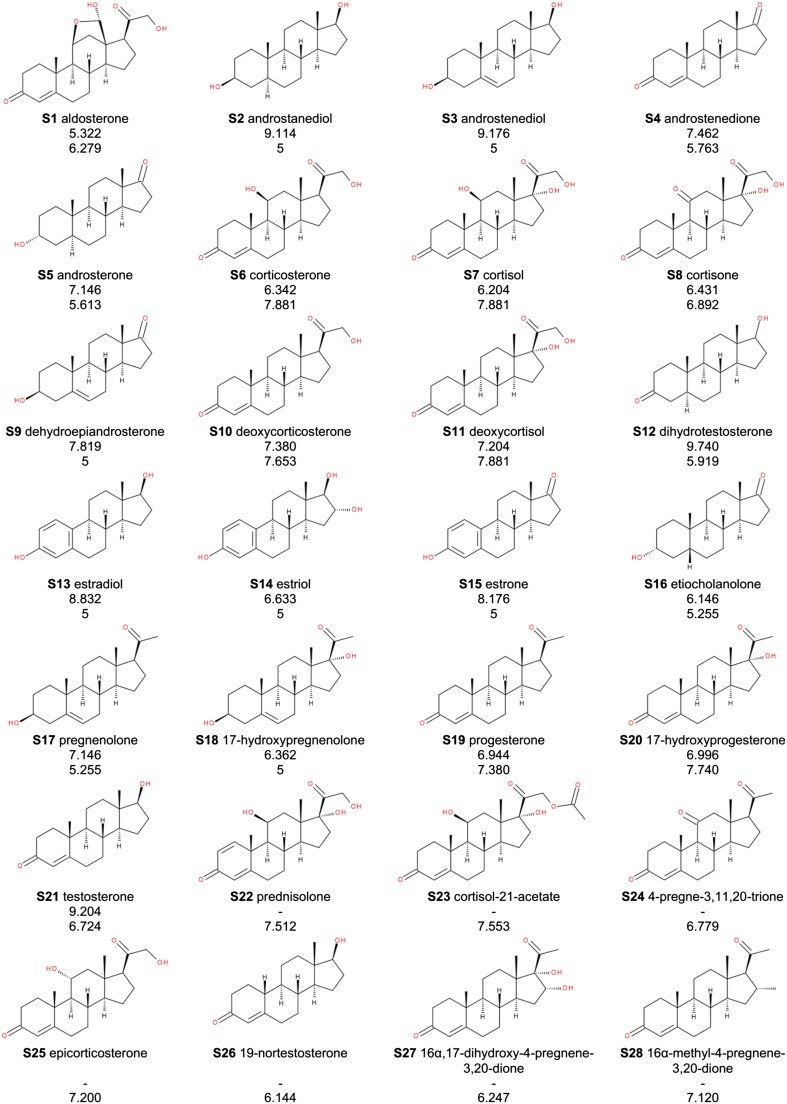

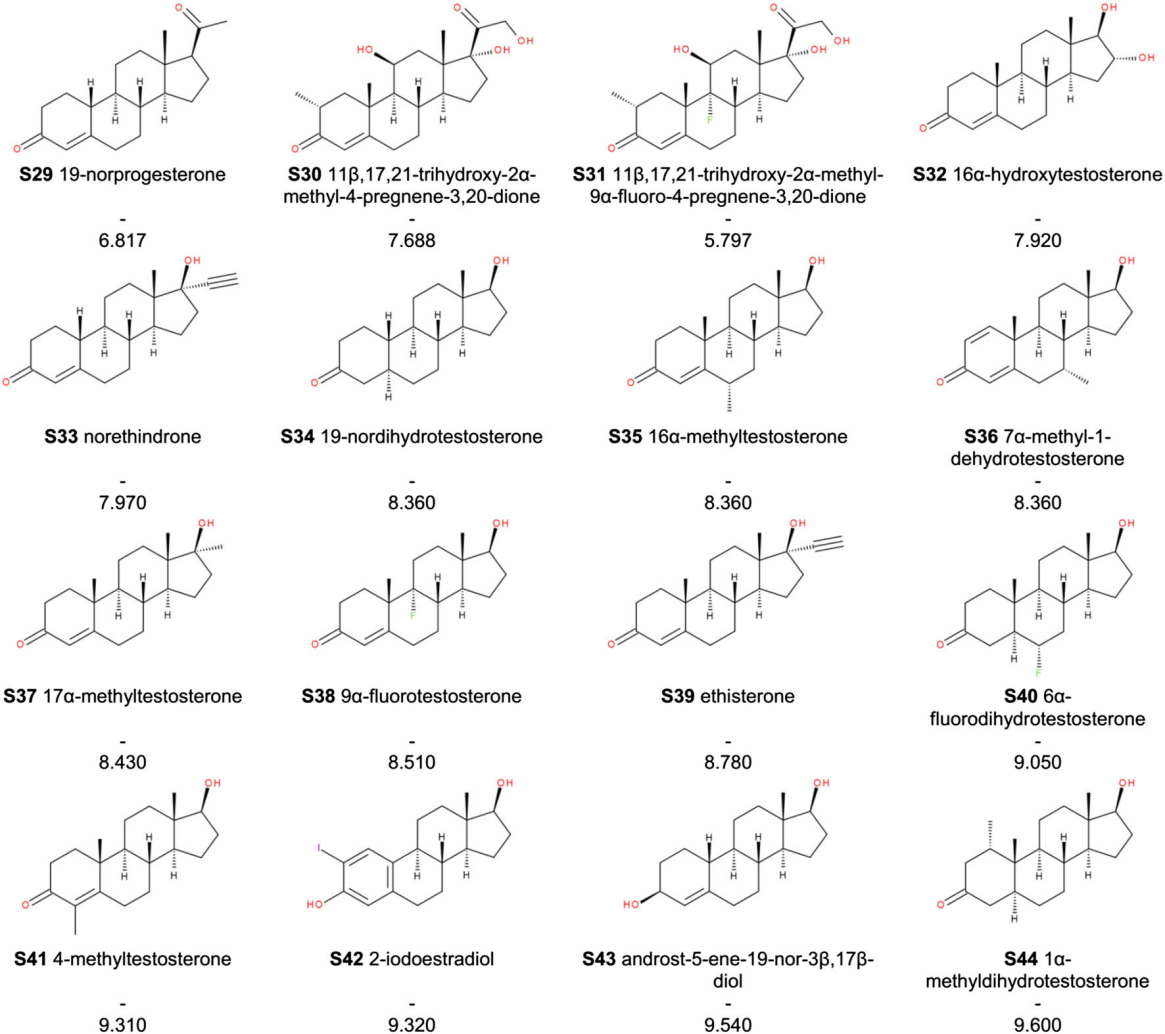
Steroid molecules used in Cases I and II. The figures under names are binding affinities to corticosteroid-binding globulin (*upper figures*, Case II) and sex-hormone-binding globulin (*lower figures*, Case I)

In QSAR studies, the Cramer data set of 21 steroids (Fig. 2) binding to SHBG became a benchmark set for validating novel QSAR methodologies or descriptors (Cramer et al. 1988; Coats 1998). Therefore, it is a good point for illustrating the danger of chance correlations.

In our study, we trained QSAR models of up to 3 independent variables, using 89 2D molecular descriptors. The top 10 models are presented in Table 2. Their statistical parameters are not the best ones, but they could be perceived as acceptable by some QSAR modellers (*r* ^2^ = 0.762–0.811, *q* ^2^ = 0.613–0.706). On the other hand, the equations are physically uninterpretable as almost all descriptors (except for P_VSA_s_4 and NsssCH) cannot be translated (at least without great effort) into the language of atoms, functional groups or other chemical structures. Still, many authors ‘interpret’ similar models just by providing brief descriptions of how the descriptors are calculated and conclude that the equation(s) could serve for screening chemical libraries in search of new active compounds.

**Table 2:** Top 10 QSAR models obtained by the GFA procedure (Case I)

No	*K*_aff_ =	*r* ^2^	*q* ^2^	*R* ^2^	${}^{c}R_{p}^{2}$	$r_{{m \left( {\text{test}} \right)}}^{2}$
1	−69.185 − 1.103 × X5v + 38.155 × MATS5m + 44.524 × SpMin3_Bh(m)	0.811	0.690	0.051, *n* = 10	0.339	0.023
2	−60.380 + 28.860 × MATS5m − 45.301 × MATS3v + 34.208 × SpMin3_Bh(m)	0.792	0.706	0.015, *n* = 9	0.312	<0.001
3	−89.817 + 160.850 × X2A + 36.696 × MATS5m + 30.049 × SpMin3_Bh(m)	0.784	0.701	0.027, *n* = 12	0.300	0.017
4	22.870 + 127.78 × VE2_Dt − 5.549 × SpDiam_AEA(dm) − 0.559 × NsssCH	0.782	0.673	0.322, *n* = 8	0.297	0.289
5	14.787 − 2.262 × IDDE + 157.890 × VE2_Dt − 3.801 × SpDiam_AEA(dm)	0.777	0.669	0.030, *n* = 8	0.290	0.024
6	−25.180 + 30.414 × MATS5 m + 23.871 × SpMin3_Bh(m) − 1.935 × SpDiam_AEA(dm)	0.771	0.679	0.146, *n* = 9	0.280	0.097
7	2.426 + 186.700 × VE2_Dt + 0.070 × P_VSA_s_4 − 4.433 × SpDiam_AEA(dm)	0.766	0.620	0.062, *n* = 10	0.273	0.059
8	−67.765 + 34.796 × MATS5m − 8.129 × MATS8 m + 40.696 × SpMin3_Bh(m)	0.766	0.615	0.1085, *n* = 11	0.273	0.084
9	61.058 + 170.730 × VE2_Dt − 178.400 × ChiA_B(p) − 5.089 × SpDiam_AEA(dm)	0.765	0.613	0.057, *n* = 6	0.271	0.050
10	35.620 + 34.296 × MATS5m − 25.263 × SpMax4_Bh(i) + 32.296 × SpMin3_Bh(m)	0.762	0.633	0.016, *n* = 10	0.266	0.003

*K*_aff_ logarithm of the affinity for SHBG, *ChiA_B(p)* average Randic-like index from Burden matrix weighted by polarizability, *IDDE* mean information content on the distance degree equality, *MATS3v* Moran autocorrelation of lag 3 weighted by van der Waals volume, *MATS5m* Moran autocorrelation of lag 5 weighted by mass, *MATS5m* Moran autocorrelation of lag 5 weighted by mass, *MATS8m* Moran autocorrelation of lag 8 weighted by mass, *NsssCH* Number of atoms of type sssCH, *P_VSA_s_4* P_VSA-like on I-state, bin 4, *SpDiam_AEA(dm)* spectral diameter from augmented edge adjacency matrix weighted by dipole moment, *SpMax4_Bh(i)* largest eigenvalue *n*. 4 of Burden matrix weighted by ionization potential, *SpMin3_Bh(m)* smallest eigenvalue *n*. 3 of Burden matrix weighted by mass, *VE2_Dt* average coefficient of the last eigenvector from detour matrix, *X2A* average connectivity index of order 2, *X5v* valence connectivity index of order 5

*r* ^2^ coefficient of determination in the training set

*q* ^2^ cross-validated coefficient of determination in the training set (internal validation)

*R* ^2^ coefficient of determination in the test set (external validation)

*n* number of molecules used for the external validation (that is: found in the applicability domain of a given model)

${}^{c}R_{p}^{2}$ a parameter of model non-randomness proposed in (Mitra et al. 2010)

$r_{{m \left( {\text{test}} \right)}}^{2}$ a parameter describing the prediction of the absolute response date of the test set, proposed in (Pratim Roy et al. 2009)

The moderately optimistic *r* ^2^ and *q* ^2^ become not optimistic at all if one looks at the outcomes of the models trained on *y*-scrambled activity data or those trained on pseudo-descriptors (Table 3). It turns out that none of the obtained ‘real’ models is better than the 99th percentile (+2.3 SD) of the models found in the *y*-randomization or pseudo-descriptors tests (mhr^2^ + 2.3 SD of models trained on pseudo-descriptors is as high as 0.825). Further, external validation on several ligands (6–12, depending on the applicability domain of a given model, (Tables 2 and SI-2) extracted from the extended steroid set (Cherkasov et al. 2008) yields very poor results, with the coefficient of determination in the test set (*R* ^2^) not higher than 0.270.

**Table 3:** Predictive power of the chance models (Case I)

	mhr^2^	SD	+1 SD	+2.3 SD	+3 SD	mhq^2^	SD	+2.3 SD	+3 SD
*y*-scrambling	0.544	0.123	0.667	0.827	0.913	0.316	0.183	0.737	0.865
Pseudo-descriptors (original distributions)	0.656	0.066	0.723	0.809	0.855	0.546	0.101	0.778	0.849
Pseudo-descriptors (uniform distributions)	0.669	0.068	0.737	0.825	0.873	0.571	0.091	0.780	0.843

*mhr* ^2^ mean highest coefficient of determination from 300 test runs

*SD* standard deviation

*mhq* ^2^ mean highest cross-validated coefficient of determination from 300 test runs

The models in Table 2 are thus: internally quite good but uninterpretable and not better than random models. As such, they could be expected to have poor predictive power, what is then shown in external validation (Tables 2 and SI-2).

### Case II

In the second of the studied cases, we used the same Cramer steroid data set (Cramer et al. 1988; Coats 1998), but this time the target property was binding affinity for the corticosteroid-binding globulin (CBG). CBG is another steroid transporting protein, but contrary to SHBG, it binds preferentially corticosteroids and progestogens, while androgens or oestrogens have only moderate affinity for it (Rosner 1990). The protein is implicated in the inflammatory response by modulating the corticosteroid concentration at the site of inflammation (Klieber et al. 2007). On the other hand, under physiological conditions it buffers blood cortisol levels. In CBG-deficient individuals observed are symptoms of extreme tiredness, hypotension or chronic muscle pain (Marathe and Torpy 2012; Torpy et al. 2013). Some research has been also made on the role of CBG in glucose metabolism (Fernández-Real et al. 1999), obesity (Ousova et al. 2004) or sperm motility (Teves et al. 2010). Recently, an interesting proposition was put forward to use engineered CBGs as drug delivery agents (Chan et al. 2014).

In the study, we divided the CBG set into training and test subsets (in proportion 21:10). The GFA procedure was used to find equations of up to 3 variables, using 49 descriptors derived from 2D and 3D molecular structures. The top 10 models are presented in Table 4.

**Table 4:** Top 10 QSAR models obtained by the GFA procedure (Case II)

No	*K*_aff_ =	*r* ^2^	$q_{{ ( {\text{LOO)}}}}^{2}$	$q_{{ ( {\text{L3O)}}}}^{2}$	*R* ^2^	${}^{c}R_{p}^{2}$	$r_{{m \left( {\text{test}} \right)}}^{2}$
1	−9.394 + 5.644 × *q*2 − 0.145 × ALogP_MR + 10.252 × JX	0.892	0.823	0.826	0.365, *n* = 7	0.546	0.131
2	−9.934 − 5.780 × q3 + 2.055 × Shadow_Ylength − 0.263 × Shadow_YZ	0.885	0.802	0.739	0.325, *n* = 8	0.538	0.178
3	−4.871 − 6.046 × q3 + 5.795 × JX − 1.168 × Shadow_Zlength	0.879	0.805	0.786	0.732, *n* = 8	0.531	0.630
4	8.117 − 6.065 × q3 + 1.013 × CHI_3_C − 2.035 × Shadow_Zlength	0.877	0.790	0.778	0.635, *n* = 8	0.529	0.366
5	−1.520 − 6.636 × q3 + 0.840 × Shadow_Ylength − 1.209 × Shadow_Zlength	0.877	0.775	0.750	0.255, *n* = 7	0.529	0.078
6	4.437 + 1.986 × q2 − 5.312 × q3 − 1.112 × Shadow_Zlength	0.871	0.803	0.802	0.654, *n* = 7	0.522	0.484
7	9.151 − 6.785 × q3 + 2.103 × srcm2 − 2.035 × Shadow_Zlength	0.867	0.789	0.736	0.691, *n* = 8	0.518	0.371
8	3.079 + 2.976 × q2 − 4.246 × q3 − 0.074 × ALogP_MR	0.867	0.788	0.800	0.619, *n* = 8	0.518	0.425
9	3.451 − 2.334 × q1 − 5.156 × q3 − 1.034 × Shadow_Zlength	0.866	0.805	0.102	0.579, *n* = 8	0.516	0.334
10	−5.392 − 6.237 × q3 − 0.076 × ALogP_MR + 1.113 × Shadow_Ylength	0.863	0.694	0.600	0.636, *n* = 8	0.513	0.514

*K*_aff_ negative logarithm of the affinity for CBG, *ALogP_MR* the Ghose and Crippen estimate of molar refractivity, *CHI_3_C* Kier and Hall molecular connectivity index, cluster subgraph of order 3, *JX* Balaban Index JX, *q1* CHELPG atomic charge at the C1 atom of the steroid skeleton (IUPAC numbering), *q2* CHELPG atomic charge at the C2 atom of the steroid skeleton (IUPAC numbering), *q3* CHELPG atomic charge at the C3 atom of the steroid skeleton (IUPAC numbering), *Shadow_Ylength* length of molecule in the y dimension, *Shadow_YZ* area of the molecular shadow in the yz plane, *Shadow_Zlength* length of molecule in the *z* dimension, *srcm2* Sinister-Rectus Chirality Measure weighted by mass

*r* ^2^ coefficient of determination in the training set

$q_{{ ( {\text{LOO)}}}}^{2}$ cross-validated coefficient of determination in the training set (internal validation, leave-one-out procedure)

$q_{{ ( {\text{L3O)}}}}^{2}$ cross-validated coefficient of determination in the training set (internal validation, leave-three-out procedure)

*R* ^2^ coefficient of determination in the test set (external validation)

*n* number of molecules used for the external validation (that is: found in the applicability domain of a given model)

${}^{c}R_{p}^{2}$ a parameter of model non-randomness proposed in (Mitra et al. 2010)

$r_{{m \left( {\text{test}} \right)}}^{2}$ a parameter describing the prediction of the absolute response date of the test set, proposed in (Pratim Roy et al. 2009)

The presented models have good statistical parameters (*r* ^2^ = 0.863–0.892, $q_{(LOO)}^{2}$ = 0.694–0.823, $q_{(L3O)}^{2}$ = 0.600–0.826). A look at the performance of the chance models allows to conclude that in this modelling situation (21 data points and 49 molecular descriptors) the probability of chance correlations is lower than in the Case I (Table 5) . All obtained QSAR models are significantly better than *y*-scrambled or pseudo-descriptor models. Their additional advantage is clear physical meaning of the variables used (except for two topological descriptors). External validation on several ligands (7–8, depending on the applicability domain of a given model, Tables 4 and SI-3) yields both poor and good results. Three models have external *R* ^2^ much lower than 0.5, but on the other hand in the case of the best two (model 3 and 7) the value is 0.732 and 0.691, which is a decent outcome. Model 3 fulfils also the widely accepted criteria for QSAR model predictive power (Golbraikh and Tropsha 2002): *q* ^2^ > 0.5, *R* ^2^ > 0.6, $\frac{{R^{2} - R_{0}^{2} }}{{R^{2} }} < 0.1$ and 0.85 ≤ *k* ≤ 1.15, where $R_{0}^{2}$ denotes external coefficient of determination forced through the origin, and *k* is a slope of the regression line through the origin. Here, the value of $r_{{m \left( {\text{test}} \right)}}^{2}$ parameter is 0.630 and this further supports the predictive power of the model with regard to exact affinity values of the test compounds. Note also that the model has good $R_{\text{int}}^{2}$ and $Q_{\text{int}}^{2}$ metrics (their values provided in Table SI-4 in Electronic Supporting Material).

**Table 5:** Predictive power of the chance models (Case II)

	mhr^2^	SD	+1 SD	+2.3 SD	+3 SD	mhq^2^	SD	+2.3 SD	+3 SD
*y*-scrambling	0.475	0.105	0.580	0.716	0.789	0.341	0.148	0.681	0.785
Pseudo-descriptors (original distributions)	0.570	0.090	0.660	0.776	0.839	0.456	0.118	0.728	0.811
Pseudo-descriptors (uniform distributions)	0.558	0.098	0.656	0.784	0.853	0.431	0.128	0.725	0.815

*mhr* ^2^ mean highest coefficient of determination from 300 test runs

*SD* standard deviation

*mhq* ^2^ mean highest cross-validated coefficient of determination from 300 test runs

**Fig. 3:**
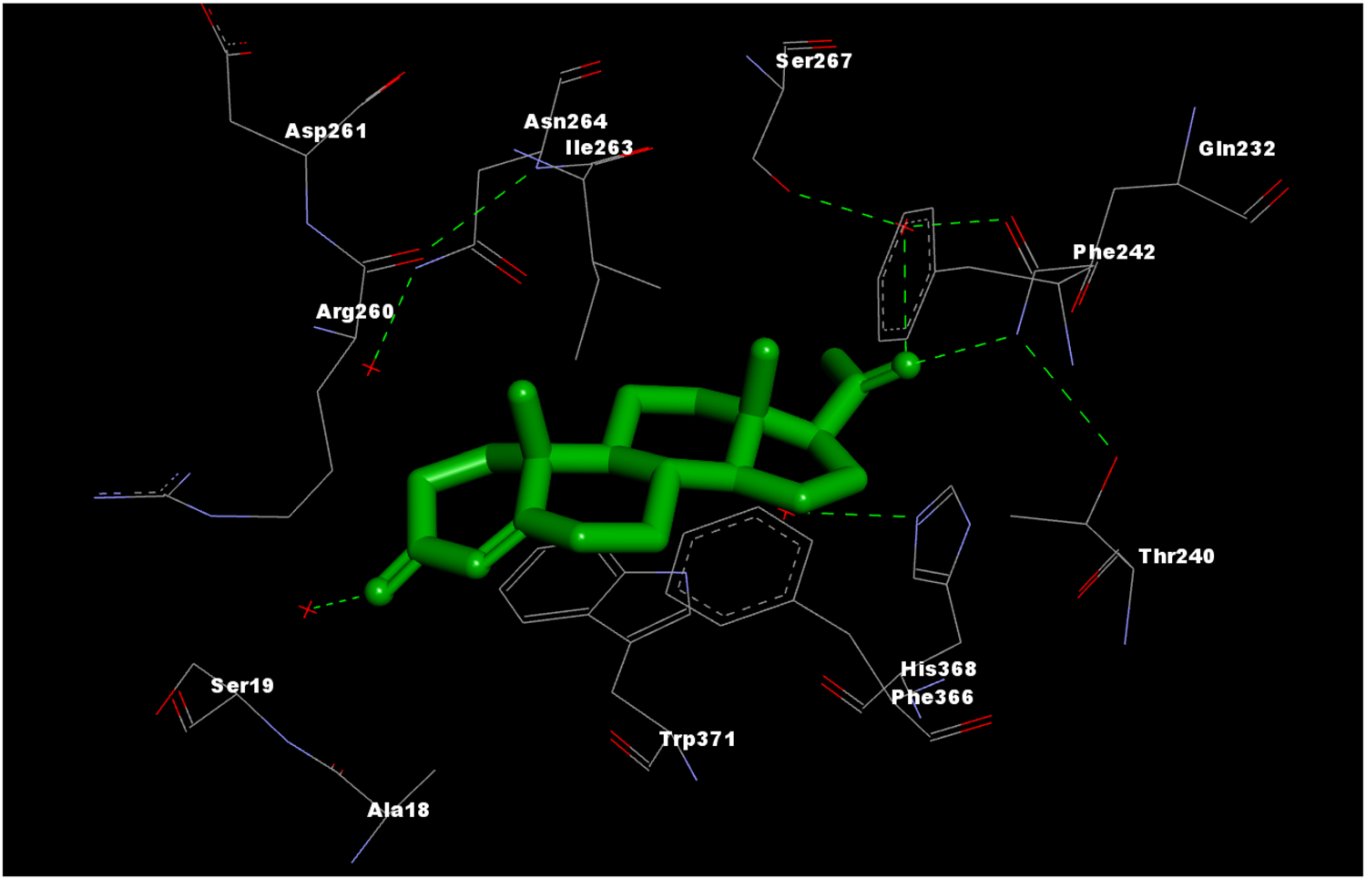
Progesterone in the binding site of CBG (PDB accession code: 4BB2)

**Fig. 4:**
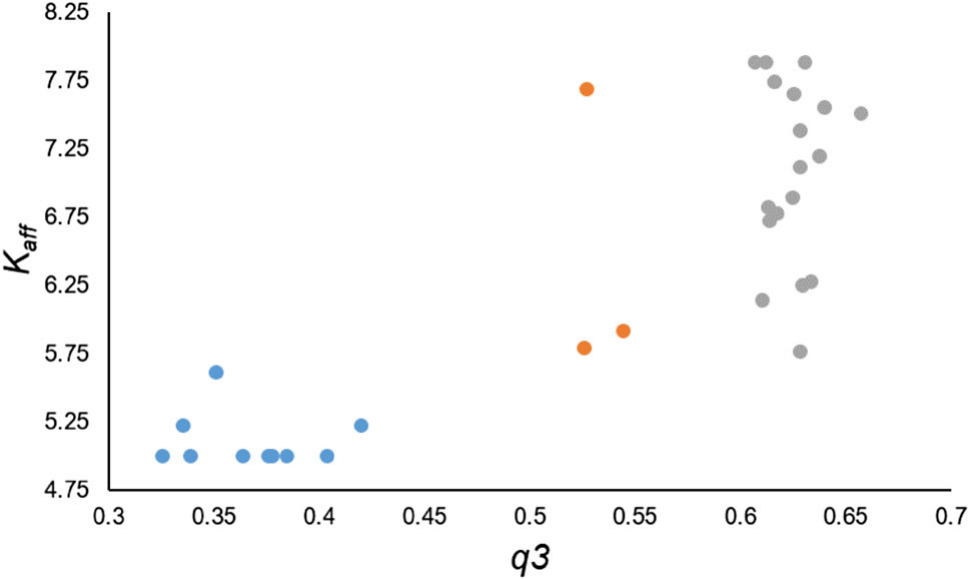
Plot of *q*3 and *K*_aff_

Experimental structures of the corticosteroid-binding globulin co-crystallized with cortisol or progesterone (Fig. 3. PDB accession codes: 2V95, 4BB2) allow to interpret the models in structural terms (Klieber et al. 2007; Gardill et al. 2012). The interaction of corticosteroids or progesterone with CBG depends mainly on hydrogen bonds formed by polar functions at C and D steroidal rings (IUPAC steroid nomenclature). Although in our models, no charge descriptors for C- and D-rings atoms are present, this is accounted for by shape descriptors like Shadow_Zlength or srcm2. The presence or absence of pharmacophoric polar elements (C17 chain with a keto group, C11 hydroxyl group etc.) affects the size of the molecule or non-superposability on its mirror image and thus these important features are indirectly included into equations. On the other hand, *q*3 descriptor depicts electrostatics of the A ring. If we plot *q*3 and *K*_aff_, there appear three clusters (Fig. 4). The lowest *q*3 values characterize molecules with a hydroxyl group attached to C3 atom. The middle three are those with C3-keto group but with the charge modified due to a C2-substituent or saturation of the C4–C5 double bond (dihydrotestosterone). The third cluster contains molecules with C3-keto group. There exists some rough correlation between *q*3 and *K*_aff_ (*r* ^2^ = 0.690) showing that the C3-keto group (with its geometry and electrostatics) is preferred over C3-hydroxyl, perhaps due to a formation of more favourable hydrogen bonds network with water and surrounding amino acids of the binding site. The clustering achieved by *q*3 is refined by the shape descriptors (bearing also indirectly information on the most important pharmacophoric elements) or the topological JX descriptor (the role of which is not easily interpretable on its own) and thus good QSAR models are obtained.

Concluding, the models obtained in Case II are not only internally good, but also significantly better than chance correlations in this modelling situation. Further, they are well-interpretable. As such, they may be expected to possess some predictive power, what is shown by external validation.

### Case III

Case III represents a different modelling situation than the previous two, since it was attempted to build Fujita-Ban models (Fujita and Ban 1971). This type of QSAR analysis uses variables that are discrete indicators (taking 0 or 1 values) of presence or absence of particular structural elements in a molecule. Fujita-Ban models have a clear physical sense, but on the other hand they contain multiple parameters. The ratio of the number of equation variables to the number of data points is usually larger than in ‘typical’ QSARs with variables of a continuous character.

In this Case, we considered a group of 36 active (training set) and 10 inactive (test set) fentanyl derivatives (3-methyl-1,4-disubstituted piperidines) (Lalinde et al. 1990) (Table SI-1). Fentanyls or more basically 4-anilidopiperidines are one of the most important groups of analgesics. Since the discovery of fentanyl in the late 1950s (Janssen et al. 1963), numerous derivatives with varying activity have been synthesized and described (Vardanyan and Hruby 2014). Four of them are present in medicinal practice and these are fentanyl, alfentanil, sufentanil and remifentanil. They are used for pain management in terminally ill cancer patients and anaesthesia. Fentanyls act at the µ-opioid receptor (MOR), belonging to the family A of G-protein coupled receptors (GPCR). Unfortunately, this class of analgesics is not free of typical unwanted side effects of opioids (Chaney 1995) nor of their potential for abuse (Skulska et al. 2005; Algren et al. 2013; Mounteney et al. 2016).

**Fig. 5:**
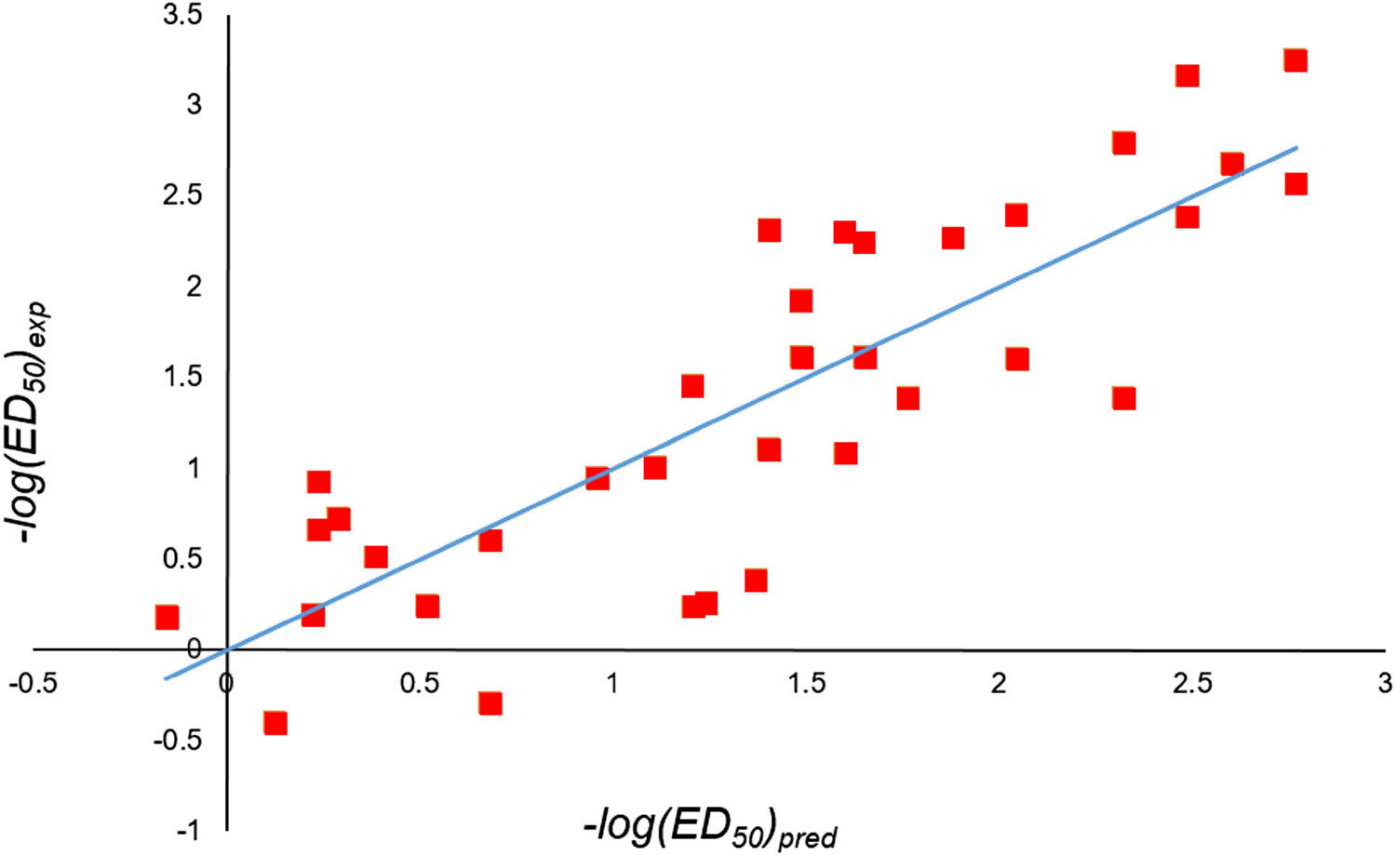
Plot of predicted and experimental activities for the QSAR model in Case III

The dependent variable for QSAR model building was the effective dose ED_50_ in mouse hot plate test (analgesic activity test). Multiple Linear Regression correlated indicator variables with the activity to give an equation the terms of which are presented in Table 6. The plot of experimental vs predicted activities is given in Fig. 5. The equation has a moderate *r* ^2^ of 0.718 and large errors of terms coefficients, rendering a few of the terms insignificant. On the other hand the predictive power of chance models in this particular modelling situation is rather low, and even such moderately good QSAR model is better than the best predictions trained on random data (Table 7). Large errors may be attributed to inaccuracies of the experimental data (in vivo testing), but still the model is able to predict inactivity of six of 10 compounds not used in model training. In the case of the remaining four, it predicts low or very low activity (Table SI-1).

**Table 6:** Fujita-Ban QSAR model of fentanyls activity (Case III)

	Equation terms	Coefficient	Standard error
3-CH_3_	Intercept (parent)	2.604	0.330
	Cis/trans	−0.001	0.225
L	Phenylethyl	−0.281	0.237
	Tetrazolylethyl	−1.657	0.413
	Thienylethyl	0.000	0.000
R	CH_2_OCH_3_	0.000	0.000
	CH(CH_3_)OCH_3_	−1.116	0.262
	Furoyl	−0.722	0.247
X	F	0.163	0.262
	Cl	−0.918	0.348

**Table 7:** Predictive power of the chance models (Case III)

	mhr^2^	SD	+1 SD	+2.3 SD	+3 SD	mhq^2^	SD	+2.3 SD	+3 SD
Pseudo-descriptors test (original distributions)	0.293	0.108	0.401	0.541	0.617	–	–	–	–
Pseudo-descriptors test (uniform distributions)	0.285	0.112	0.397	0.542	0.621	–	–	–	–
*y*-scrambling	0.235	0.092	0.327	0.446	0.511	–	–	–	–

Most *q* ^2^ were negative

*mhr* ^2^ mean highest coefficient of determination from 300 test runs

*SD* standard deviation

*mhq* ^2^ mean highest cross-validated coefficient of determination from 300 test runs

As to the model interpretability, it must be said that statistical insignificance of the terms causes any interpretations to be only rough in their nature, even though all terms are physically well-defined. Nevertheless, the coefficients of L-descriptors (Table 6) seem to fit the Structure-Activity Relationship knowledge on fentanyl derivatives, with the following order of L-substitution preference: thienylethyl (as in sufentanil) > phenylethyl (as in fentanyl) > tetrazolylethyl (as in alfentanil) (Volpe et al. 2011). Regarding the R-part of the molecules, it is clearly visible that R-methoxymethyl is more favourable for analgesic activity than its branched (R–CH(CH_3_)OCH_3_ **)** or rigidified (R-furoyl) counterparts. The freedom of rotation and lack of steric hindrance may allow more facile formation of hydrogen bonds. Unfortunately, the role of 3-Me stereochemistry is not well rendered in the model by the statistically insignificant coefficient. In general, however, it is well-known that 3-*cis* substituents are more active (Vuckovic et al. 2009). No clear conclusions may be drawn about X substituents, again due to the insignificance of the coefficients.

The model presented in Case III is most probably not a random one, but still it is rather inaccurate. As mentioned, large coefficient errors are attributable to the inaccuracies of in vivo data. Thus, even though the model is not random and partially interpretable, it may be of only partial utility.

### Case IV

In the last Case, the objective was to create a classification model able to discern glucocorticoid receptor (GR) binders and non-binders. GR is a nuclear receptor-binding corticosteroid and acts as a transcription factor to up- or downregulate the expression of certain genes (Luisi et al. 1991; Yudt and Cidlowski 2002). It is involved in maintaining homeostasis by affecting inflammatory responses, cellular proliferation and differentiation in target tissues (Funder 1997). GR ligands include classical steroidal glucocorticoids which are used for tackling diseases involving inflammation (van der Velden 1998; Barnes 1998), for immunosuppression (Coutinho and Chapman 2011) or for cancer treatment (Coleman 1992; Vaidya et al. 2010). Current medicinal chemistry focuses on development of selective glucocorticoid receptor modulators (based on scaffolds different from the steroidal), which would be void of typical side effects of steroidal glucocorticoids (De Bosscher 2010).

**Fig. 6:**
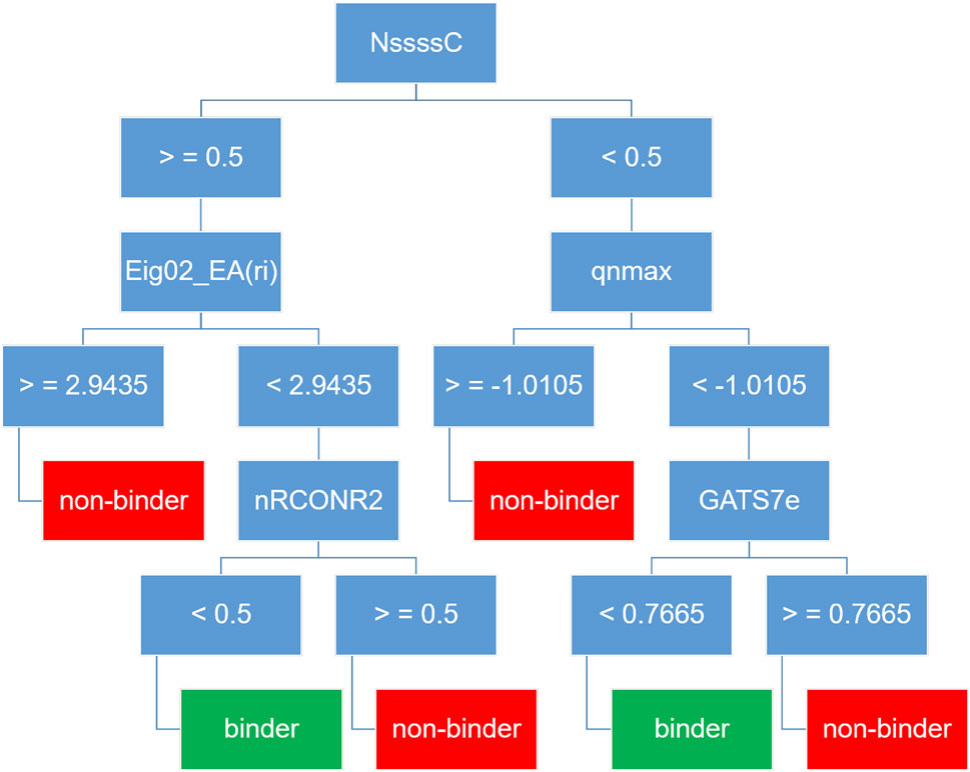
Scheme of the classification tree (Case IV). *Eig02_EA(ri)* eigenvalue *n*. 2 from edge adjacency matrix weighted by resonance integral, *GATS7e* Geary autocorrelation of lag 7 weighted by Sanderson electronegativity, *nRCONR2* number of tertiary amides (aliphatic), *NssssC* number of atoms of type ssssC (>C<), where < or > are two single bonds, *qnmax* maximum negative charge

For the modelling purposes, we decided to mimic a most common real-world situation (as for example in virtual screening experiments), where the number of receptor binders is much smaller than that of non-binders. Therefore, we decided to keep the original proportion of actives vs decoys occurring in the DUD-E data set (Mysinger et al. 2012) that is 1:36. The machine learning algorithm obtained the classification model presented in Fig. 6. It is a simple decision tree with a maximal node depth being three. The model has good statistical parameters of internal predictions (Table 8). Models trained on random data have significantly lower accuracies, precisions and specificities and significantly higher fall-out rates, but on the other hand they are comparably sensitive. F1-score, a measure considering both precision and sensitivity, is however much better for the model trained on true data. The quality of the decision tree may also be assessed by comparison to no-model predictions: ‘all binders’, ‘all non-binders’ or ‘coin-toss’. The analysis of their parameters (Table 8) gives optimistic results, with precision and F1-score again much better in the case of the model trained on true data.

**Table 8:** Statistical parameters of the decision tree (Case IV) and comparison with different random models and no-model predictions

	TP^a^	TN^b^	FP^c^	FN^d^	ACC^e^	PREC^f^	SENS^g^	SPEC^h^	FALL^i^	F1^j^
Training set										
QSAR model trained on real data										
	90	3371	226	10	0.94	0.28	0.90	0.94	0.06	0.43
No-model: all binders										
	100	0	3600	0	0.03	0.03	1.00	0.00	1.00	0.06
No-model: all non-binders										
	0	3600	0	100	0.97	NaN^k^	0.00	1.00	0.00	NaN
No-model: coin toss										
	50	1800	1800	50	0.50	0.03	0.50	0.50	0.50	0.06
QSAR models trained on pseudo-descriptors (original distributions)										
Mean	80.00	1785.40	1811.60	19.00	0.50	0.04	0.81	0.50	0.50	0.08
SD^l^	9.20	402.30	402.30	9.20	0.11	0.01	0.09	0.11	0.11	0.02
+1 SD	89.20	2187.80	2213.90	28.20	0.61	0.05	0.90	0.61	0.62	0.10
+2.3 SD	101.20	2710.80	2736.90	40.20	0.75	0.06	1.02	0.75	0.76	0.13
QSAR models trained on pseudo-descriptors (uniform distribution)										
Mean	82.30	1666.20	1930.80	16.70	0.47	0.04	0.83	0.46	0.54	0.08
SD	13.50	461.70	461.70	13.50	0.12	0.01	0.14	0.13	0.13	0.02
+1 SD	95.80	2127.90	2392.60	30.30	0.60	0.05	0.97	0.59	0.67	0.10
+2.3 SD	113.40	2728.10	2992.80	47.90	0.75	0.06	1.15	0.76	0.83	0.13
Test set										
QSAR model trained on real data										
Mean	45	1674	126	5	0.93	0.26	0.90	0.93	0.07	0.40

^a^True positives

^b^True negatives

^c^False positives

^d^False negatives

^e^Accuracy

^f^Precision

^g^Sensitivity

^h^Specificity

^i^Fall-out

^j^F1-score

^k^Not a number

^l^Standard deviation

Regarding the interpretability of the model, it must be concluded that even though some of the descriptors used in the model are physically well understandable, the tree does not allow to provide explicit statements about what structural features are important for GR binding. The model is thence uninterpretable. Still, when applied for classification of the test set containing 1850 molecules (50 binders and 1800 decoys), it performs correctly for about 93% of cases. The precision (0.26) and F1-score (0.40) are here similar to the ones for the internal predictivity.

Thus, the classification tree in the Case IV is both internally good as well as better than random. The model is not easily interpretable, however physical interpretability is not what is usually expected of classification models. The most important here is good predictivity, what is shown in external validation.

## Conclusions

Since the danger of overfitting QSAR models, when working on large descriptor pools, is very high, it is desirable to perform tests showing the performance of models built on random data. In this study we introduce a simple software tool SCRAMBLE’N’GAMBLE that is aimed at facilitating data preparation for *y*-scrambling and pseudo-descriptors tests. As shown in the Cases studied in the paper, these tests may be applied to all sorts of QSAR techniques, including both classical linear equations, Fujita-Ban models or classification trees. Their results indicate what the quality of a studied model is like in comparison to chance models obtained from random data. While the non-randomness is not the ultimate hallmark of QSAR models’ possible utility, it is a good practice to consider it along with internal statistical parameters and interpretability of the model. On the other hand, if a model performs no better than chance, it is very probable that it will not be of any use in predicting activities of novel compounds. SCRAMBLE’N’GAMBLE (available for free at: http://www.drugdesign.pl/scramble-n-gamble/) is hoped to help QSAR researchers to perform y-scrambling and pseudo-descriptors testing.

## Electronic supplementary material

Below is the link to the electronic supplementary material.

Supplementary material 1 (PDF 364 kb)

Supplementary material 2 (TXT 1 kb)

Supplementary material 3 (TXT 0 kb)

Supplementary material 4 (TXT 49 kb)

Supplementary material 5 (TXT 24 kb)
