# Supplementary material for: SCRAMBLE’N’GAMBLE: a tool for fast and facile generation of random data for statistical evaluation of QSAR models
Source: Chem Zvesti. 2017 Jun 5;71(11):2217–32. doi: 10.1007/s11696-017-0215-7 (PMC5655615; doi:10.1007/s11696-017-0215-7)
Supplement: Supplementary file 1 — Supplementary material 1 (PDF 364 kb) [file 11696_2017_215_MOESM1_ESM.pdf]

ELECTRONIC SUPPORTING MATERIAL TO:

SCRAMBLE'N'GAMBLE – a tool for fast and facile generation of random data for statistical evaluation of QSAR models

Piotr F. J. Lipiński[1] • Przemysław Szurmak[2]

✉ Piotr F. J. Lipiński

plipin@icm.edu.pl

[1] Department of Neuropeptides, Mossakowski Medical Research Centre Polish Academy of Sciences, 02-106 Warszawa, Poland. E-mail: plipin@icm.edu.pl.

[2] ChemPharmSoft, 01-926 Warszawa, Poland

Tables of contents:

Table SI-1 Fentanyl derivatives used in Case III: indicator variables and the activity data. 2

Table SI-2 Results of validation for the Case I 4

Table SI-3 Results of validation for the Case II 5

Table SI-4 R^2_{int} and Q^2_{int} metrics for Case II models. 6

Tables SI-5 – SI-8 are given in separate txt files.

Full Gaussian 09 Citation 7

Table SI-1 Fentanyl derivatives used in Case III: indicator variables and the activity data.

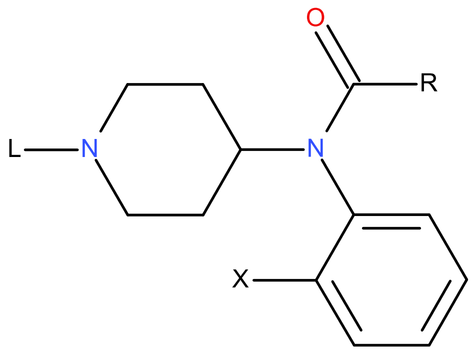

	3-CH_3	L			R			X			experimental		predicted
**cmpd**	**cis/trans**	**phenylethyl**	**tetrazolylethyl**	**thienylethyl**	**CH_2OCH_3**	**$CH(CH_3)OCH_3$**	**furoyl**	**F**	**Cl**	**OCH_3**	**ED_{50}**	**-log(ED_{50})**	**-log(ED_{50})**
**F34**	1	1	0	0	1	0	0	0	0	0	0.0016	2.80	2.32
**F35**	0	1	0	0	1	0	0	0	0	0	0.041	1.39	2.32
**F37**	1	0	0	1	1	0	0	0	0	0	0.0021	2.68	2.60
**F38**	1	1	0	0	1	0	0	1	0	0	0.0041	2.39	2.49
**F39**	0	1	0	0	1	0	0	1	0	0	0.00069	3.16	2.49
**F40**	1	0	1	0	1	0	0	1	0	0	0.098	1.01	1.11
**F42**	1	0	0	1	1	0	0	1	0	0	0.00056	3.25	2.77
**F43**	0	0	0	1	1	0	0	1	0	0	0.0027	2.57	2.77
**F44**	1	1	0	0	1	0	0	0	0	1	0.1125	0.95	0.96
**F46**	1	0	0	1	1	0	0	0	0	1	0.547	0.26	1.24
**F47**	1	1	0	0	1	0	0	0	1	0	0.078	1.11	1.40
**F48**	0	1	0	0	1	0	0	0	1	0	0.00486	2.31	1.40
**F49**	1	1	0	0	0	1	0	0	0	0	0.035	1.46	1.21
**F50**	0	1	0	0	0	1	0	0	0	0	0.575	0.24	1.21
**F53**	1	0	0	1	0	1	0	0	0	0	0.0119	1.92	1.49
**F54**	0	0	0	1	0	1	0	0	0	0	0.0244	1.61	1.49
**F55**	1	1	0	0	0	1	0	1	0	0	0.41	0.39	1.37
**F57**	1	0	0	1	0	1	0	1	0	0	0.0057	2.24	1.65

**F58**	0	0	0	1	0	1	0	1	0	0	0.0244	1.61	1.65
**F60**	0	1	0	0	0	1	0	0	0	1	0.651	0.19	-0.15
**F62**	1	0	0	1	0	1	0	0	0	1	2.5	-0.40	0.13
**F63**	1	1	0	0	0	1	0	0	0	1	0.669	0.17	-0.16
**F64**	0	1	0	0	0	1	0	0	1	0	0.188	0.73	0.29
**F65**	1	1	0	0	0	0	1	0	0	0	0.005	2.30	1.60
**F66**	0	1	0	0	0	0	1	0	0	0	0.082	1.09	1.60
**F67**	1	0	1	0	0	0	1	0	0	0	0.638	0.20	0.22
**F68**	1	0	0	1	0	0	1	0	0	0	0.0054	2.27	1.88
**F69**	1	1	0	0	0	0	1	1	0	0	0.041	1.39	1.76
**F70**	1	0	1	0	0	0	1	1	0	0	0.305	0.52	0.39
**F72**	1	0	0	1	0	0	1	1	0	0	0.004	2.40	2.04
**F73**	0	0	0	1	0	0	1	1	0	0	0.025	1.60	2.05
**F74**	1	1	0	0	0	0	1	0	0	1	0.217	0.66	0.24
**F75**	0	1	0	0	0	0	1	0	0	1	0.118	0.93	0.24
**F77**	1	0	0	1	0	0	1	0	0	1	0.568	0.25	0.52
**F78**	1	1	0	0	0	0	1	0	1	0	1.96	-0.29	0.68
**F79**	0	1	0	0	0	0	1	0	1	0	0.247	0.61	0.68
**inactive compounds not used for training Fujita-Ban QSAR model**													
**F36**	1	0	1	0	1	0	0	0	0	0	inactive	inactive	0.95
**F41**	0	0	1	0	1	0	0	1	0	0	inactive	inactive	1.11
**F45**	1	0	1	0	1	0	0	0	0	1	inactive	inactive	-0.42
**F51**	1	0	1	0	0	1	0	0	0	0	inactive	inactive	-0.17
**F52**	0	0	1	0	0	1	0	0	0	0	inactive	inactive	-0.17
**F56**	0	0	1	0	0	1	0	1	0	0	inactive	inactive	-0.01
**F59**	1	1	0	0	0	1	0	0	0	1	inactive	inactive	-0.16
**F61**	1	0	1	0	0	1	0	0	0	1	inactive	inactive	-1.53
**F71**	0	0	1	0	0	0	1	1	0	0	inactive	inactive	0.39
**F76**	1	0	1	0	0	0	1	0	0	1	inactive	inactive	-1.14

Table SI-2 Results of validation for the Case I

cmpd	experimental activity	activity calculated from equation: (Table 2)									
		**1**	**2**	**3**	**4**	**5**	**6**	**7**	**8**	**9**	**10**
**S33**	7.97	7.79	6.94	7.31	6.32	7.04	7.11	5.65	8.12	6.80	7.60
**S34**	8.36	8.36	8.76	8.69	-	-	-	6.62	8.91	-	8.38
**S35**	8.36	7.37	7.61	7.58	7.61	8.66	7.66	8.44	6.69	8.36	6.45
**S36**	8.36	8.49	8.75	8.58	7.79	8.37	8.38	10.25	7.95	9.28	7.81
**S38**	8.51	-	-	5.85	7.84	7.58	6.33	11.61	6.44	-	6.61
**S39**	8.78	8.41	7.93	7.83	5.98	5.15	7.80	4.95	8.40	5.96	8.21
**S40**	9.05	-	8.11	7.33	7.66	9.08	7.68	11.63	7.32	9.11	7.20
**S41**	9.31	7.00	6.67	6.49	8.29	8.40	7.25	8.05	6.69	8.41	6.72
**S42**	9.32	8.70	7.50	8.77	8.64	7.83	8.97	11.72	8.72	-	-
**S43**	9.54	8.79	8.41	8.66	-	-	-	7.29	9.19	-	8.83
**S44**	9.60	7.26	7.58	7.54	-	-	8.06	-	8.62	-	7.23

Table SI-3 Results of validation for the Case II

cmpd	experimental activity	activity calculated from equation: (Table 3)									
		1	2	3	4	5	6	7	8	9	10
**S1**	-6.28	-	-6.48	-	-6.84	-6.77	-6.95	-6.52	-7.41	-6.85	-7.20
**S6**	-7.88	-7.47	-7.18	-7.18	-7.31	-7.17	-7.20	-7.08	-7.28	-7.26	-7.22
**S12**	-5.92	-5.89	-5.99	-6.37	-6.05	-6.19	-6.07	-6.12	-5.91	-6.16	-6.10
**S13**	-5.00	-5.02	-4.95	-4.49	-	-	-	-	-5.24	-	-4.82
**S16**	-5.23	-6.21	-7.33	-	-	-	-	-	-5.56	-	-5.25
**S17**	-5.23	-6.61	-5.20	-5.19	-4.93	-5.13	-5.01	-5.03	-5.52	-5.03	-5.68
**S21**	-6.72	-5.78	-6.84	-6.77	-6.94	-6.99	-6.79	-6.87	-6.42	-6.86	-6.69
**S23**	-7.55	-	-	-7.87	-6.95	-	-7.57	-7.24	-	-7.53	-
**S27**	-6.25	-7.23	-7.63	-7.12	-6.94	-7.43	-7.27	-6.94	-7.34	-7.31	-7.54
**S30**	-7.69	-	-	-6.83	-6.84	-6.17	-	-6.87	-	-6.82	-

Table SI-4 R^2_{int} and Q^2_{int} metrics for Case II models.

**No**	K_{aff} =	R^2_{int}	Q^2_{int}
1	-9.394 + 5.644 * *q2* − 0.145 * *ALogP_MR* + 10.252 * *JX*	0.140	0.007
2	-9.934 − 5.780 * *q3* + 2.055 * *Shadow_Ylength* − 0.263 * *Shadow_YZ*	0.183	<0.001
3	-4.871 − 6.046 * *q3* + 5.795 * *JX* − 1.168 * *Shadow_Zlength*	0.130	0.001
4	8.117 − 6.065 * *q3* + 1.013 * *CHI_3_C* − 2.035 * *Shadow_Zlength*	0.111	<0.001
5	-1.520 − 6.636 * *q3* + 0.840 * *Shadow_Ylength* − 1.209 * *Shadow_Zlength*	0.128	<0.001
6	4.437 + 1.986 * *q2* − 5.312 * *q3* − 1.112 * *Shadow_Zlength*	0.127	0.005
7	9.151 − 6.785 * *q3* + 2.103 * *srcm2* − 2.035 * *Shadow_Zlength*	0.107	0.001
8	3.079 + 2.976 * *q2* − 4.246 * *q3* − 0.074 * *ALogP_MR*	0.159	0.014
9	3.451 − 2.334 * *q1* − 5.156 * *q3* − 1.034 * *Shadow_Zlength*	0.514	<0.001
10	-5.392 − 6.237 * *q3* − 0.076 * *ALogP_MR* + 1.113 * *Shadow_Ylength*	0.140	<0.001

Full Gaussian 09 citation

Gaussian 09, Revision D.01, Frisch, M. J.; Trucks, G. W.; Schlegel, H. B.; Scuseria, G. E.; Robb, M. A.; Cheeseman, J. R.; Scalmani, G.; Barone, V.; Mennucci, B.; Petersson, G. A.; Nakatsuji, H.; Caricato, M.; Li, X.; Hratchian, H. P.; Izmaylov, A. F.; Bloino, J.; Zheng, G.; Sonnenberg, J. L.; Hada, M.; Ehara, M.; Toyota, K.; Fukuda, R.; Hasegawa, J.; Ishida, M.; Nakajima, T.; Honda, Y.; Kitao, O.; Nakai, H.; Vreven, T.; Montgomery, J. A., Jr.; Peralta, J. E.; Ogliaro, F.; Bearpark, M.; Heyd, J. J.; Brothers, E.; Kudin, K. N.; Staroverov, V. N.; Kobayashi, R.; Normand, J.; Raghavachari, K.; Rendell, A.; Burant, J. C.; Iyengar, S. S.; Tomasi, J.; Cossi, M.; Rega, N.; Millam, J. M.; Klene, M.; Knox, J. E.; Cross, J. B.; Bakken, V.; Adamo, C.; Jaramillo, J.; Gomperts, R.; Stratmann, R. E.; Yazyev, O.; Austin, A. J.; Cammi, R.; Pomelli, C.; Ochterski, J. W.; Martin, R. L.; Morokuma, K.; Zakrzewski, V. G.; Voth, G. A.; Salvador, P.; Dannenberg, J. J.; Dapprich, S.; Daniels, A. D.; Farkas, Ö.; Foresman, J. B.; Ortiz, J. V.; Cioslowski, J.; Fox, D. J. Gaussian, Inc., Wallingford CT, **2009**.
